# Transformation of a Silent Adrencorticotrophic Pituitary Tumor Into Central Nervous System Melanoma

**DOI:** 10.1177/2324709613494008

**Published:** 2013-06-17

**Authors:** Brandon A. Miller, Tomoko Tanaka, Adriana G. Ioachimescu, Cristina Vincentelli, Christina L. Appin, Nelson M. Oyesiku

**Affiliations:** 1 Emory University School of Medicine, Atlanta, GA, USA

**Keywords:** central nervous system melanoma, pituitary carcinoma, pituitary adenoma

## Abstract

Silent adrenocorticotrophic pituitary adenomas are nonfunctioning pituitary adenomas that express adrenocorticotrophic hormone (ACTH) but do not cause the clinical or laboratory features of hypercortisolemia. Primary central nervous system (CNS) melanoma is well documented, but rarely originates in the sellar region or pituitary gland. Here we report transformation of an aggressive silent adrenocorticotrophic pituitary adenoma that transformed into CNS melanoma and review other presentations of pituitary melanoma. A 37-year-old woman initially presented with apoplexy and an invasive nonfunctioning pituitary macroadenoma for which she underwent transphenoidal surgery. The patient underwent 3 subsequent surgeries as the tumor continued to progress. Pathology from the first 3 operations showed pituitary adenoma or carcinoma. Pathology from the final surgery showed melanoma and the magnetic resonance imaging characteristics of the tumor had changed to become consistent with CNS melanoma. Dermatologic and ophthalmologic examinations did not identify cutaneous or ocular melanoma. The patient’s disease progressed despite aggressive surgical, medical and radiologic treatment. To our knowledge, this is the first report demonstrating transformation of a primary pituitary tumor into melanoma. The mechanism of tumor transformation is unclear, but it is possible that a mutation in the original ACTH-producing tumor lead to increased cleavage of pro-opiomelanocortin or ACTH into α-melanocyte-stimulating hormone, which in turn stimulated the expression of microopthalmia transcription factor, leading to melanocytic phenotype transformation.

## Clinical Case

A 37-year-old woman with no significant medical history presented to an outside hospital with acute headache and vision loss. Magnetic resonance imaging (MRI) showed a pituitary macroadenoma with invasion of the cavernous sinus, compression of the optic chiasm, and a cystic posterior component. Endocrine evaluation at the time of diagnosis was consistent with a nonfunctioning tumor. The patient underwent emergent transphenoidal surgery and subtotal resection. Pathological diagnosis was pituitary adenoma. Five years later, the patient underwent fractionated radiosurgery for tumor progression in the right cavernous sinus. Subsequently, her MRIs remained stable; however, the patient developed hypopituitarism.

**Figure 1.**
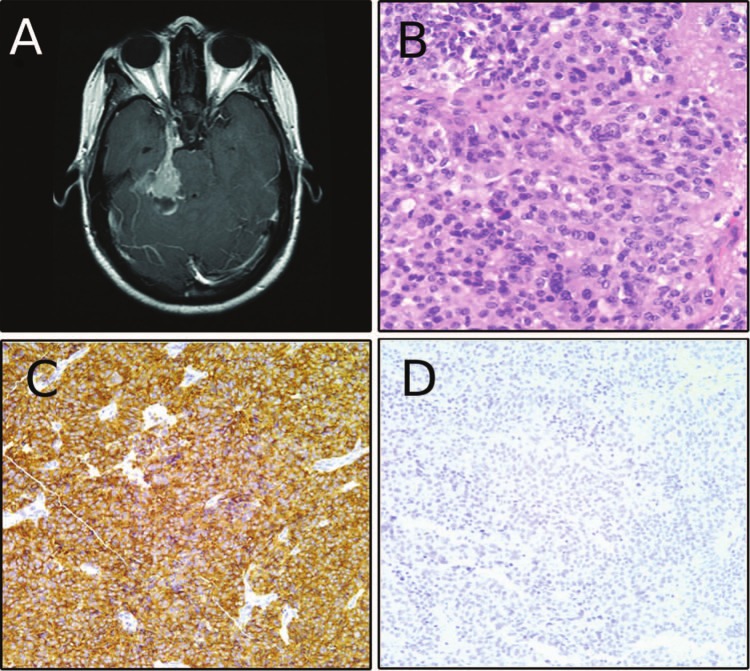
(A) Preoperative magnetic resonance image before the patient’s first surgery at our institution showing contrast-enhancing cavernous sinus-based lesion. (B) Hematoxylin and esosin staining consistent with pituitary adenoma. (C) Positive synaptophysin labeling. (D) Absence of microopthalmia transcription factor labeling.

The patient was initially referred to our clinic at 45 years of age. At that time, MRI showed an enhancing mass in the right cerebellopontine angle and cavernous sinus, without residual tumor in the sella (Figure 1A). On follow-up studies, the tumor progressed along the tentorium and cerebellopontine angle. Consequently, at age 47, the patient underwent right petrosal and subtemporal craniotomy with subtotal resection. Pathology was again consistent with pituitary adenoma (Figure 1B) with positive synaptophysin and adrenocorticotrophic hormone (ACTH) immunoreactivity (Figure 1C, ACTH not shown). Tissue from this surgery was later stained for the melanoma markers microopthalmia transcription factor (MITF) and S100 and was negative for both (Figure 1D, S100 not shown). One year later, MRI revealed stable cavernous sinus disease and new contrast-enhancing lesions at the previous craniotomy site, cerebellar tonsils, and C2. The patient was treated with intensity-modulated radiation therapy and follow-up MRI showed further cystic transformation of the cavernous sinus tumor but no progression of the other lesions.

**Figure 2.**
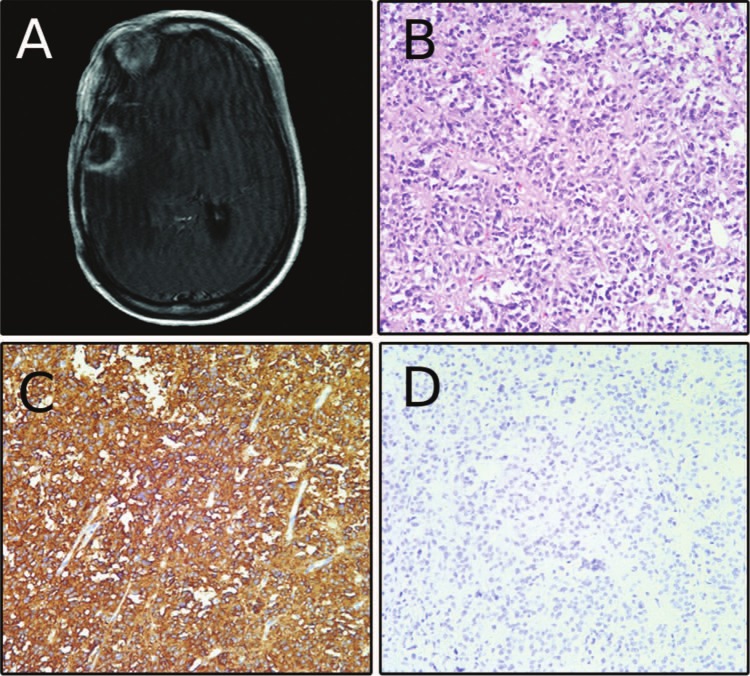
(A) Preoperative magnetic resonance image before the patient’s second surgery at our institution showing contrast-enhancing dural-based lesion. (B) Hematoxylin and eosin staining consistent with pituitary adenoma. (C) Positive synaptophysin labeling. (D) Absence of microopthalmia transcription factor labeling.

At age 49 years, MRI showed tumor progression in the posterior fossa, along the tentorium, right frontoparietal, and right temporal lobes. The patient underwent radiosurgery to the right temporal and right frontoparietal lesions, Six months after radiosurgery, the patient presented to our emergency room with left-sided weakness. MRI showed a contrast-enhancing right temporal dural-based lesion with extensive edema and mass effect (Figure 2A). A craniotomy was performed just anterior to the previous surgical site and the tumor and surrounding dura were excised. Hematoxylin and eosin staining was consistent with pituitary adenoma, with similar morphology to the previous specimen (Figure 2B). The MIB1 proliferation index was 2% to 3%, and synaptophysin and ACTH immunoreactivity were again positive (Figure 2C, ACTH not shown). As with the previous specimen, the tumor was negative for the melanoma markers MITF and S100 (Figure 2C, S100 not shown).

**Figure 3.**
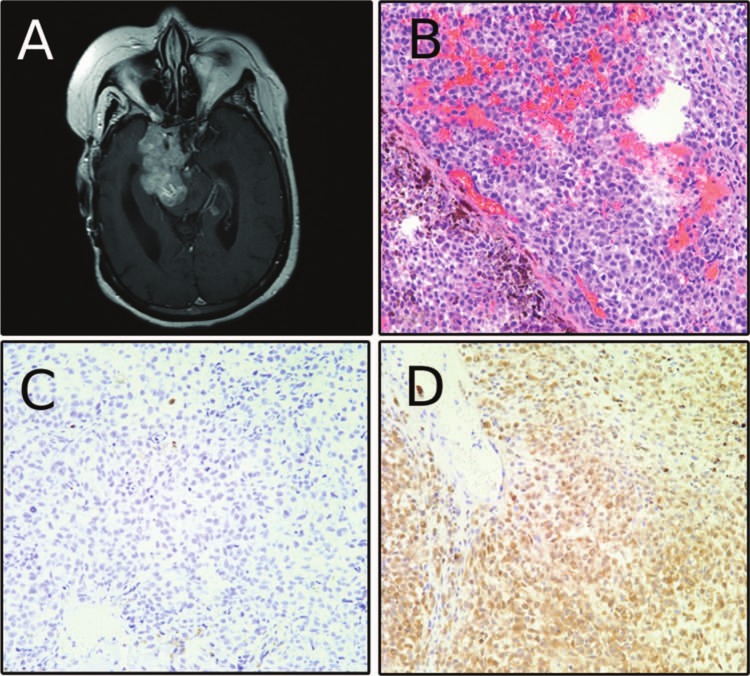
(A) Preoperative magnetic resonance image before the patient’s third surgery at our institution showing contrast-enhancing cavernous sinus-based lesion. (B) Hematoxylin and eosin staining consistent with melanoma. (C) Absence of synaptophysin labeling. (D) Positive microopthalmia transcription factor labeling.

**Figure 4.**
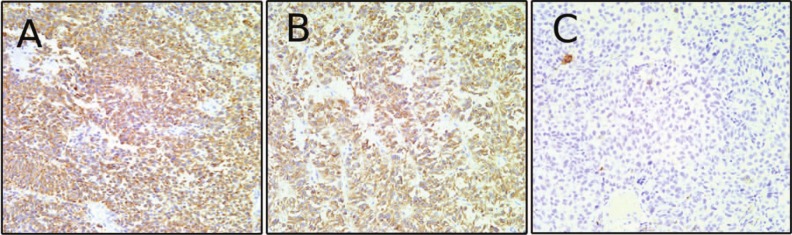
(A) Positive pankeratin AE1/AE3 labeling from specimen from first surgery at our institution. (B) Positive pankeratin AE1/AE3 labeling from specimen from second surgery at our institution. (A) Negative pankeratin AE1/AE3 labeling from specimen from third surgery at our institution.

**Figure 5.**
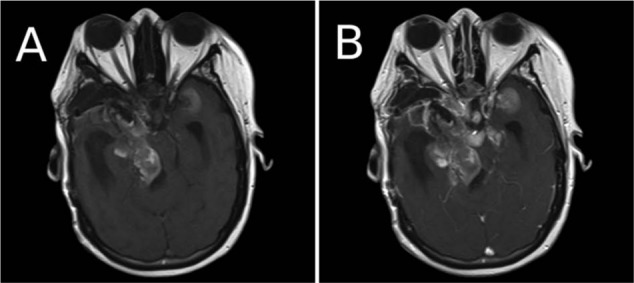
Final magnetic resonance image after the third surgery at our institution. (A) Precontrast T1 image with bright signal typical of central nervous system melanoma. (B) Postcontrast magnetic resonance image showing extensive tumor spread.

The patient continued to be followed in our clinic by neurosurgery, endocrinology and radiation oncology. She was maintained on cortisol and levothyroxine replacement and her ACTH levels ranged between 6 and 17 pg/mL (normal levels = 5-27 pg/mL). At age 51, she complained of double vision and facial numbness, and MRI showed progression of the tumor in the cavernous sinus and along the tentorium with compression of the right cerebral peduncle. She was initiated on carbergoline based on a case report of a silent corticotroph tumor responding to this dopamine agonist.^1^ One month later, MRI revealed further tumor progression, and cabergoline was discontinued. Temozolomide, which has also been shown to be effective against pituitary adenomas^2^ was then initiated. After 2 courses of temozolomide, the patient developed left lower extremity weakness, right facial weakness, and hearing loss. MRI showed extensive parasellar disease encasing the right internal carotid artery, entering the posterior fossa, jugular foramen, internal auditory canal, foramen magnum, and along the gyrus rectus (Figure 3A). At this point, the tumor had become hyperintense on T1 precontrast imaging. For the first time, ACTH became higher than normal (31 ng/mL). A frontotemporal craniotomy was preformed for tumor debulking. Intraoperatively, the tumor appeared dark, vascular, and friable. Pathology showed a high nuclear to cytoplasmic ratio and extensive melanin deposition, consistent with malignant melanoma (Figure 3B). Imunohistochemistry was negative for synaptophysin (Figure 3C) and positive for MITF and S100 (Figure 3D, S100 not shown), confirming the diagnosis of melanoma. The tumor at this time no longer expressed synaptophysin or pankeratin AE1/AE3, markers of pituitary adenomas that had been present in the previous 2 tumors (Figure 4). After the final surgery, the patient underwent a full dermatologic and ophthalmologic exam that showed no melanotic lesions. Postoperatively, the patient had a prolonged intensive care unit course and slow neurological recovery. Additional chemotherapy was not instituted because of the patient’s poor functional status. MRI 2 months after the final surgery showed extensive tumor progression throughout the brain and meninges that was both contrast enhancing and T1 bright, unlike the patient’s original lesion that was only contrast enhancing (Figure 5). After discussion with the family, the patient was transferred to hospice care and died at age 51.

## Discussion

Silent adrenocorticotrophic pituitary adenomas (SCAs) were first described by Kovacs et al^3^ in 1978 as pituitary adenomas that produce ACTH but do not cause hypercortisolemia. In our case, pathology evaluation initially supported the diagnosis of SCA. Based on the imaging characteristics during the 6 years of follow-up at our institution, the tumor transformed into a silent corticotroph carcinoma. Although this progression has been previously reported,^4^ there are no reports of SCAs transforming into melanoma or other tumor phenotypes.

Primary central nervous system (CNS) melanoma is rare, especially in the sellar region.^5^ This patient had 3 pathological diagnoses all showing pituitary adenoma or carcinoma, 2 of which, when analyzed at our institution, showed positive ACTH immunoreactivity and no immunoreactivity for melanoma markers. Therefore, there is no doubt that the patient’s original CNS disease was of pituitary origin. Interestingly, as the patient underwent repeat MRI scans, lesions were detected at the foramen magnum and within the upper cervical spinal canal, which are sites of melanin production in the CNS. On the first MRI in which these lesions appeared, they were seen to be contrast enhancing but T1 dark, as was the patient’s original parasellar disease. On the patient’s final MRI, these lesions were T1 bright, as well as contrast enhancing, again, like the patient’s parasellar disease. Unfortunately, no specimens were available from these lesions, so the histology or possible histological change of these lesions is unknown. Regardless, the parasellar disease, which was resected during the first and final operations at our institution, clearly underwent transformation between those surgeries. Imaging supports the hypothesis that all the patient’s tumor burden underwent similar transformation.

The possibility that this lesion was a collision tumor of 2 different pathologies at the same location is unlikely. Although there are reports of collision tumors in the pituitary region,^6,7^ the incidence of collision tumors in the sella is less than 2%.^8^ In the series by Koutourousiou et al,^8^ all the collision tumors were composed of pituitary adenomas with other tumors or lesions of the CNS such as another pituitary tumor, Rathke’s cleft cyst, sarcoid, or gangliocytoma. There is no report that we are aware of describing CNS melanoma and a pituitary tumor occurring together. The patient underwent complete dermatologic and ophthalmologic exams after her final surgery, neither of which revealed any melanotic lesions. Therefore, the primary melanotic lesion would have to have been extracutaneous, which is rare.^9^ Additionally, the radiographic transformation of the tumor from a T1 dark to T1 bright, and the loss of all pituitary adenoma markers in the final pathological specimen support a diagnosis of tumor transformation rather than collision.

There are previous reports of CNS tumors exhibiting melanocytic differentiation. Several CNS tumor types, including astrocytomas,^10^ glioblastomas,^11^ and ependymomas^12^ have all been shown to rarely express melanin. However, these tumors still retained their previous phenotype even as they expressed melanin. This is the first report of a pituitary tumor undergoing melanocytic differentiation and losing its expression of its previous antigens.

Cases of melanoma metastatic to the pituitary have been reported, though this is a rare occurrence.^7^ Reports of primary melanoma of the sellar region are even rarer.^13-16^ In these cases of isolated pituitary melanoma, the cellular site of origin has been hypothesized to be the meninges,^14^ a Rathke’s cyst,^13^ or the pituitary gland itself.^17^ In these cases, like ours, there were no melanotic lesions found outside the CNS. However, no prior description of primary pituitary melanoma involved transformation of one tumor phenotype to another, as was the case in the patient we describe.

The exact mechanism of this tumor’s transformation is unclear. This patient received multiple rounds of radiation therapy, which may have predisposed the tumor to additional mutations. The mechanism of tumor transformation likely involves the processing of the ACTH. ACTH is derived from pro-opiomelanocortin (POMC) and is further processed in the pituitary into α-melanocyte stimulating hormone (α-MSH), which also results from POMC cleavage. α-MSH induces skin pigmentation,^18^ binds to extracellular receptors, and induces MITF expression, a key transcription factor for melanocyte differentiation and survival.^19^ It is possible that a mutation in the original ACTH-producing tumor lead to increased cleavage of POMC or ACTH into α-MSH, which in turn stimulated the expression of MITF, leading to melanocytic phenotype transformation. It has been shown that nonfunctional macroadenomas produce increased levels of ACTH precursors.^20^ This would magnify the effect of POMC being cleaved to form α-MSH. Regardless of the mechanism, the potential of a SCA to transform into melanoma demonstrates the aggressive behavior of SCAs and reveals a unique origin of pituitary melanoma.

## References

[1] Petrossians P Ronci N Valdés Socin H ACTH silent adenoma shrinking under cabergoline. Eur J Endocrinol. 2001;144:51-57. 11174837 10.1530/eje.0.1440051

[2] Marucci G. Treatment of pituitary neoplasms with temozolomide: a review. Cancer. 2011;117:4101-4102. 21387270 10.1002/cncr.26000

[3] Kovacs K Horvath E Bayley TA Hassaram ST Ezrin C Silent corticotroph cell adenoma with lysosomal accumulation and crinophagy. A distinct clinicopathologic entity. Am J Med. 1978;64:492-499. 76447 10.1016/0002-9343(78)90236-x

[4] Brown RL Wollman R Weiss RE Transformation of a pituitary macroadenoma into to a corticotropin-secreting carcinoma over 16 years. Endocr Pract. 2007;13:463-471. 17872347 10.4158/EP.13.5.463

[5] Rousseau A Bernier M Kujas M Varlet P Primary intracranial melanocytic tumor simulating pituitary macroadenoma: case report and review of the literature. Neurosurgery. 2005;57:E369. 16094139 10.1227/01.neu.0000166686.19823.a9

[6] Leung GK Chow WS Tan KC Fan YW Lam KS Metastatic melanoma of the pituitary gland. Case report. J Neurosurg. 2003;99:913-915. 14609173 10.3171/jns.2003.99.5.0913

[7] Wang YY Norris A du Plessis D Gnanalingham KK Melanoma of the sellar region. J Clin Neurosci. 2011;18:154-156. 20965730 10.1016/j.jocn.2010.07.111

[8] Koutourousiou M Kontogeorgos G Wesseling P Grotenhuis AJ Seretis A Collision sellar lesions: experience with eight cases and review of the literature. Pituitary. 2010;13:8-17. 19551516 10.1007/s11102-009-0190-2 PMC2807600

[9] Hussein MR Extracutaneous malignant melanomas. Cancer Invest. 2008;26:516-534. 18568775 10.1080/07357900701781762

[10] Kanzawa T Takahashi H Hayano M Mori S Shimbo Y Kitazawa T Melanotic cerebral astrocytoma: case report and literature review. Acta Neuropathol. 1997;93:200-204. 9039469 10.1007/s004010050603

[11] Jaiswal S Agrawal V Vij M Sahu RN Jaiswal AK Behari S Glioblastoma with melanotic differentiation. Clin Neuropathol. 2010;29:330-333. 20860897 10.5414/npp29330

[12] Rosenblum MK Erlandson RA Aleksic SN Budzilovich GN Melanotic ependymoma and subependymoma. Am J Surg Pathol. 1990;14:729-736. 2378394 10.1097/00000478-199008000-00005

[13] Scholtz CL Siu K Melanoma of the pituitary. Case report. J Neurosurg. 1976;45:101-103. 180267 10.3171/jns.1976.45.1.0101

[14] Tuttenberg J Fink W Back W Wenz F Schadendorf D Thome C A rare primary sellar melanoma. Case report. J Neurosurg. 2004;100:931-934. 15137611 10.3171/jns.2004.100.5.0931

[15] Aubin MJ Hardy J Comtois R Primary sellar haemorrhagic melanoma: case report and review of the literature. Br J Neurosurg. 1997;11:80-83. 9156026 10.1080/02688699746771

[16] Copeland DD Sink JD Seigler HF Primary intracranial melanoma presenting as a suprasellar tumor. Neurosurgery. 1980;6:542-545. 7413039 10.1227/00006123-198005000-00008

[17] Neilson JM Moffat AD Hypopituitarism caused by a melanoma of the pituitary gland. J Clin Pathol. 1963;16:144-149. 13937963 10.1136/jcp.16.2.144 PMC480516

[18] Bicknell AB The tissue-specific processing of pro-opiomelanocortin. J Neuroendocrinol. 2008;20:692-699. 18601691 10.1111/j.1365-2826.2008.01709.x

[19] Widlund HR Fisher DE Microphthalamia-associated transcription factor: a critical regulator of pigment cell development and survival. Oncogene. 2003;22:3035-3041. 12789278 10.1038/sj.onc.1206443

[20] Gibson S Ray DW Crosby SR Impaired processing of proopiomelanocortin in corticotroph macroadenomas. J Clin Endocrinol Metab. 1996;81:497-502. 8636257 10.1210/jcem.81.2.8636257

